# Cardiovascular Disease Prevention Education Using a Virtual Environment in Sexual-Minority Men of Color With HIV: Protocol for a Sequential, Mixed Method, Waitlist Randomized Controlled Trial

**DOI:** 10.2196/38348

**Published:** 2022-05-17

**Authors:** S Raquel Ramos, Constance Johnson, Gail Melkus, Trace Kershaw, Marya Gwadz, Harmony Reynolds, Allison Vorderstrasse

**Affiliations:** 1 School of Nursing Yale University Orange, CT United States; 2 Department of Social and Behavioral Sciences School of Public Health Yale University New Haven, CT United States; 3 Center for Interdisciplinary Research on AIDS Yale University New Haven, CT United States; 4 The Center for Drug Use and HIV Research New York University New York, NY United States; 5 Cizik School of Nursing University of Texas Houston Houston, TX United States; 6 School of Biomedical Informatics University of Texas Houston Houston, TX United States; 7 Rory Meyers College of Nursing New York University New York, NY United States; 8 Silver School of Social Work New York University New York, NY United States; 9 Department of Medicine Grossman School of Medicine New York University New York, NY United States; 10 NYU Langone Health New York, NY United States; 11 Elaine Marieb College of Nursing University of Massachusetts Amherst Amherst, MA United States

**Keywords:** virtual environment, behavioral intervention, consumer health informatics, HIV, cardiovascular disease, sexual minority men, prevention education, gamification, health communication

## Abstract

**Background:**

It is estimated that 70% of all deaths each year in the United States are due to chronic conditions. Cardiovascular disease (CVD), a chronic condition, is the leading cause of death in ethnic and racial minority males. It has been identified as the second most common cause of death in persons with HIV. By the year 2030, it is estimated that 78% of persons with HIV will be diagnosed with CVD.

**Objective:**

We propose the first technology-based virtual environment intervention to address behavioral, modifiable risk factors associated with cardiovascular and metabolic comorbidities in sexual-minority men of color with HIV.

**Methods:**

This study will be guided using social cognitive theory and the Technology Acceptance Model. A sequential, mixed method, waitlist controlled randomized control feasibility trial will be conducted. Aim 1 is to qualitatively explore perceptions of cardiovascular risk in 15 participants. Aim 2 is to conduct a waitlist controlled comparison to test if a virtual environment is feasible and acceptable for CVD prevention, based on web-based, self-assessed, behavioral, and psychosocial outcomes in 80 sexual-minority men of color with HIV.

**Results:**

The study was approved by the New York University Institutional Review Board in 2019, University of Texas Health Science Center at Houston in 2020, and by the Yale University Institutional Review Board in February 2022. As of April 2022, aim 1 data collection is 87% completed. We expect to complete data collection for aim 1 by April 30, 2022. Recruitment for aim 2 will begin mid-May 2022.

**Conclusions:**

This study will be the first online virtual environment intervention for CVD prevention in sexual-minority men of color with HIV. We anticipate that the intervention will be beneficial for CVD prevention education and building peer social supports, resulting in change or modification over time in risk behaviors for CVD.

**Trial Registration:**

ClinicalTrials.gov NCT05242952; https://clinicaltrials.gov/ct2/show/NCT05242952

**International Registered Report Identifier (IRRID):**

PRR1-10.2196/38348

## Introduction

### Background

In the United States, it is estimated that more than 70% of all deaths each year are due to chronic illness. [1,2] HIV, a blood-related chronic illness, disproportionately affects racial and ethnic minority groups, specifically African American and Latino sexual-minority men [3,4]. In alignment with the National Institutes of Health Sexual and Gender Minority Research Office, we use the words “sexual-minority men” to refer to nonheterosexual persons who identify as gay, bisexual, or queer, or persons having same-gender or same-sex attractions or behaviors.

Approximately 150 million persons are living with at least one chronic illness, and close to 30 million persons are living with 5 or more chronic conditions [5]. The chronic illness with the highest prevalence in ethnic and racial minority populations is cardiovascular disease (CVD). Cardiovascular disease is the leading cause of death in ethnic and racial minority males [6]. In 2016, it was estimated that CVD mortality among ethnic and racial minority groups was highest in African American adults. Moreover, more than 50% of Latino males aged 20 years and older had CVD. CVD risk may be lowered by following modifiable lifestyle strategies. However, HIV is an additional risk factor that increases CVD risk in African American and Latino men. Epidemiologic reports have suggested that half of African American sexual-minority men and a quarter of Latino sexual-minority men in the US will be diagnosed with HIV in their lifetime [7], demonstrating the urgency of prevention efforts in this population.

CVD has been identified as the second most common cause of death in persons with HIV, and research suggests that overall CVD awareness in the general public is inadequate [8]. By 2030, (8 years after this protocol’s publication year of 2022), it is estimated that 78% of persons with HIV will be diagnosed with CVD [9].

Cardiovascular and metabolic health disparities add to an already disproportionate HIV burden [10-13]. Persons with HIV are at higher risk of CVD than HIV-negative persons [14] as a result of chronic inflammation, certain antiretroviral therapies, and other risks, such as tobacco use [12,15-17]. Persons with HIV often lack social supports, live in low-resource communities, and have limited financial resources, exaggerating cardiovascular health disparities [18-20].

### Disparities in Accessing Health Care

Stigma, fear, and discrimination have led to underutilized health care. Previous work has substantiated some root causes of HIV health disparities as being individual- and structural-level factors [21-24]. These factors include perceived racism, sexual identity discrimination [25], and health care provider discrimination, which combine to impact treatment access and usage [21-23]. These barriers work to perpetuate silos in which sexual-minority men utilize their social networks for support and information on how to best care for their illnesses. As a result, the cumulative effects of living with HIV and not having access to equitable health care and prevention information, compounded by the increased risk of CVD, can be profoundly life threatening. If strategies and interventions to increase cardiovascular health equity in persons with HIV are not prioritized, persons with HIV will continue to experience increased morbidity, mortality, and a substantially decreased quality of life [26].

### The Promise of Prevention Education Using a Virtual Environment

Ubiquitous computing, the availability and usage of technology everywhere, has changed the paradigm of traditional in-person, face-to-face clinical interactions. Accordingly, research has shifted toward utilizing health technology as a means to promote healthy behaviors and lifestyle changes [27] via tablets, smartphones, portable trackers, and web-based interventions that use gamification [28]. Gamification using a virtual platform can increase motivation and improve user engagement, fostering health-enhancing behaviors [28,29]. Virtual environments hold much promise given their broad appeal to people of different ages, backgrounds, and levels of technological experience [30]. Research has documented the benefits and positive outcomes of using virtual environments for interventions focused on diabetes self-management [30-32], smoking cessation [33,34], HIV medication adherence [35], and prevention of risk behaviors for HIV in sexual-minority men [36,37]. They can be customized to focus on specific diseases, and content can be developed that is tailored for persons with HIV by including information on cardiovascular and metabolic disease prevention [38]. An additional benefit of using a virtual environment is anonymity. Anonymity can decrease barriers to engagement with virtual environment content and also allows individuals to speak more freely, without having to worry about someone identifying who they are [30,39]. Using an anonymous virtual environment also facilitates access to reliable prevention education and can support health-enhancing behaviors that can be transferred to real-world encounters [30]. The virtual environment can also facilitate social networking with other participants [40,41] and with health educators [37], which can build up a social support structure. This approach may prove more engaging for sexual-minority men of color, without the stigma that can be associated with face-to-face encounters [42].

### Study Objectives

Building on the Learning in a Virtual Environment (LIVE) intervention [30-32,38], we propose the first technology-based virtual intervention to address behavioral, modifiable risk factors associated with cardiovascular and metabolic comorbidities in African American and Latino sexual-minority men. Our study aims to (1) explore concerns, management, perceptions of risk, and prioritization of HIV-related comorbidities among sexual-minority men with HIV; (2) test the feasibility, acceptability, and preliminary effects of a virtual environment to address prevention of HIV-related CVD based on behavioral and psychosocial outcomes; and (2a) characterize the social network structure and usage behaviors of the participants using process data from the virtual environment. We anticipate that using a virtual environment for CVD prevention education will be advantageous to sexual-minority men of color, as it may facilitate health-promoting behaviors through education and peer social support.

## Methods

### Conceptual Framework

This study will be guided by social cognitive theory and the Technology Acceptance Model. Both models have wide usage in health communication interventions. Social cognitive theory posits that bidirectional relationships exist between person, environment, and behavior. Behaviors are learned by modeling or by observation of others [43]. We will implement modeling and observation through participant interaction with a live health educator and other peer players in the virtual environment. We anticipate the virtual environment will be used to empower participants with prevention education, skills, and increased access to social supports to facilitate health-promoting behaviors that can be translated back to activities in their daily lives for prevention of CVD. The Technology Acceptance Model is a consumer health informatics model used for determining an end user’s acceptance of technology and their intent to use a specific technology [44]. We will measure “perceived usefulness” and “perceived ease of use,” as both constructs are influential to attitudes and acceptance [45] of the virtual environment for CVD prevention education. We anticipate that if the virtual environment is useful and easy to use, this may be an antecedent to facilitating behavior change and health-promoting behaviors.

### Design

We will conduct a 2-phase, exploratory, sequential mixed methods study with a waitlist controlled clinical feasibility trial. The protocol is registered on ClinicalTrials.gov (NCT05242952) and was approved by the institutional review boards at New York University, Yale University, and the University of Texas Health Science Center at Houston.

### Eligibility

In order to meet enrollment criteria, participants must (1) be a cisgender male between ages 30 and 65; (2) identify as an ethnic or racial minority; (3) have HIV; (4) identify as gay, bisexual, or queer; (5) read and understand English; (6) have access to a computer capable of downloading and running the virtual environment software; and (7) have no cognitive impairment and no medical history of serious complications, such as myocardial infarction, congestive heart failure, coronary artery bypass graft, or cerebral vascular accident. It is documented that the highest incidence of HIV is among emerging adults (ie, up to 34 years old) [46], as chronic illness is becoming diagnosed at earlier stages of life (eg, 18 to 30 years old) with the highest prevalence at age 50 years and older [47]. Our sample age range is appropriate given the changing age demographic of chronic illness and will provide a thorough overview spanning 3 generations.

### Ethics Approval

All procedures performed in studies involving human participants are in accordance with the ethical standards of the institutional and national research committee, the 1964 Helsinki Declaration and its later amendments, or comparable ethical standards. The study was funded by the National Heart, Lung, and Blood Institute (K01HL145580) and approved by the New York University Institutional Review Board in 2019 (IRB-FY2018-2284), the University of Texas Health Science Center at Houston in 2020 (HSC-SN-20-1143), and by the Yale University Institutional Review Board in February 2022 (2000031403). Informed consent has been obtained from all individual participants who have participated thus far. We will obtain informed consent from all individuals who meet the eligibility criteria and want to participate.

### Recruitment

To increase the feasibility of participant enrollment and data collection and to use existing resources more efficiently, we will utilize a preexisting HIV registry and employ respondent-driven sampling for aims 1 and 2. Respondent-driven sampling is an ideal approach to purposively draw participants that are similar because of their connections (eg, having HIV, identifying as a sexual-minority male, and having ethnic or racial minoritized heritage), known as homophily [48-50]. This approach is fitting as a potential preemptive when recruiting historically under-sampled communities [51,52]. To prevent enrollment shortfalls, we will also partner with community-based organizations who serve LGBTQ+ populations in the New York City area. Zoom (Zoom Video Communications, Inc) will be used as the primary method of communication for enrollment, orientation to the study, and data collection in aim 1.

### Procedures

The overall purpose of this study is to prevent CVD health disparities in sexual-minority men of color with HIV using a virtual environment, which to our knowledge has not been done before. We will use the LIVE platform, a preexisting, disease-agnostic (ie, it can be modified and reused for any chronic illness) [38] virtual environment to conduct the study. To address aim 1, qualitative interviews will explore participant perceptions of health concerns. Participants will be compensated with a $30 gift card for completion of the interview. Aim 2 will be addressed in an iterative multiphase approach. In phase 1 of aim 2, beta testing will be conducted with a subset of 10 intended users to assess if the platform is embedded with relevant information and to ensure there are no technical issues. Our beta testing approach and sample size follow published recommendations [53,54]. Beta testers will receive $30 for their time. In phase 2, the LIVE platform will be used to conduct a waitlist controlled feasibility clinical trial with 80 sexual-minority men with HIV. Participants will be compensated with a $30 gift card at 3 timepoints (baseline, 3 months, and 6 months) for a total of $90 for completion of aim 2, phase 2. Subaim 2a includes collection of process data from participants. We will analyze this data to determine feasibility, usage, and acceptability. Using the LIVE platform will be advantageous to extend the utility of virtual technology to populations that are underrepresented, stigmatized by chronic illness, and at high risk for CVD. The virtual environment waitlist control clinical feasibility trial will run over a 12-month period (6 months for each group), which is an acceptable amount of time to collect preliminary data.

### Intervention Fidelity

The study staff will include the principal investigator, a research assistant, a health educator, and student volunteers. All study personnel will have completed ethical research training using the Collaborative Institutional Training Initiative online courses. We will address five components of intervention fidelity in this trial: (1) intervention design; (2) study staff training; (3) intervention delivery; (4) participant receipt of intervention; and (5) assessment of intervention outcomes. To address intervention fidelity of the study design, we have included alternative strategies for potential setbacks, such as collaborating with community-based organizations in New York City. To ensure that all study staff administer messaging and the intervention in the same manner, the principal investigator will hold training prior to the start of the intervention and periodically throughout the intervention to mitigate potential deviations from the protocol. For example, study personnel will engage in participant recruitment and enrollment roleplaying. They will practice describing the study and answering questions related to the study, and they will also be trained in how to obtain informed consent for study participation. In order to ensure standardization and replication of the intervention, we will be using standardized measures to examine outcomes. We have also developed a scope of work document that describes the processes and flow of the research-related tasks and responsibilities. It includes a script for recruiting participants. We also developed a table of contents for the live health educator sessions. This is to ensure that structured prevention education on cardiovascular health–related topics is included. Participant receipt of the intervention will be measured in aim 2 using process data, such as logins, objects clicked, time spent, areas visited, and engagement with other participants online. We will also conduct a brief postintervention assessment with a subset of participants to qualitatively assess knowledge and skills gained and applications of that knowledge and those skills to daily life.

### Orientation to the Virtual Environment

All participants enrolled in the clinical trial will receive training from the principal investigator or study personnel on how to log in to the virtual environment, create a customized avatar, and use the Zoom platform. The virtual environment will be accessed by participants through their desktop or laptop computer using an online internet connection. Participants will be given a USB headset with a microphone to plug into their home computer to allow communication and listening while engaged in the virtual environment. We recommended usage of the virtual environment at least 3 to 4 times a week. Validated self-report measures will be collected using REDCap (Research Electronic Data Capture; a online software platform created by Vanderbilt University) at baseline, 3 months, and 6 months (Table 1).

**Table 1:** Measures and outcome assessments.

Measures	Outcome assessments	Baseline	Three months	Six months
Demographics	Demographic data (age, race, ethnicity, education, and income).	✓		
Life’s Simple 7 [55]	Seven cardiovascular health metrics (blood pressure, total serum cholesterol, hemoglobin A_1c_, smoking, BMI, physical activity, and The Healthy Eating Index, scored according to 3 levels of cardiovascular health.	✓	✓	✓
Perceived Usefulness and Perceived Ease of Use [44]	Based on the Technology Acceptance Model. Outcomes include perceived usefulness (Cronbach α of .98) and perceived ease of use (Cronbach α of .94) with an online 7-point Likert scale that is modified for the virtual environment.		✓	✓
Patient Health Questionnaire-9 [56]	Nine-item reliable and valid measure of depression severity (Cronbach α of .89).	✓	✓	✓
The Revised Illness Perception Questionnaire [57]	Illness perceptions about hypertension and type 2 diabetes on a 5-point Likert scale (“strongly disagree” to “strongly agree”) (Cronbach α of 0.77 to 0.89).	✓	✓	✓
International Physical Activity Questionnaire Short Form [58-60]	Intensity of physical activity and sitting time using a 7-item, open-ended measure (Cronbach α of 0.80).	✓	✓	✓
Behavioral Risk Factors Surveillance System Questionnaire [61-63]	National behavioral risk data tool used by the Centers for Disease Control and Prevention, Health Resources and Services Administration, Veterans Administration, and the Substance Abuse and Mental Health Services Administration. Questions pertain to tobacco use and e-cigarette use (7 questions) (κ statistic of 0.81-0.92).	✓	✓	✓
Food Frequency Questionnaire [64,65]	Multifactor screener that assesses intake of fruits and vegetables, percentage of energy from fat, and intake of fiber. The screener asks respondents to report how frequently they consume foods in 16 categories. This screener has demonstrated correlations of 0.5 to 0.8 with estimated true intake.	✓	✓	✓

### Intervention

**Figure 1:**
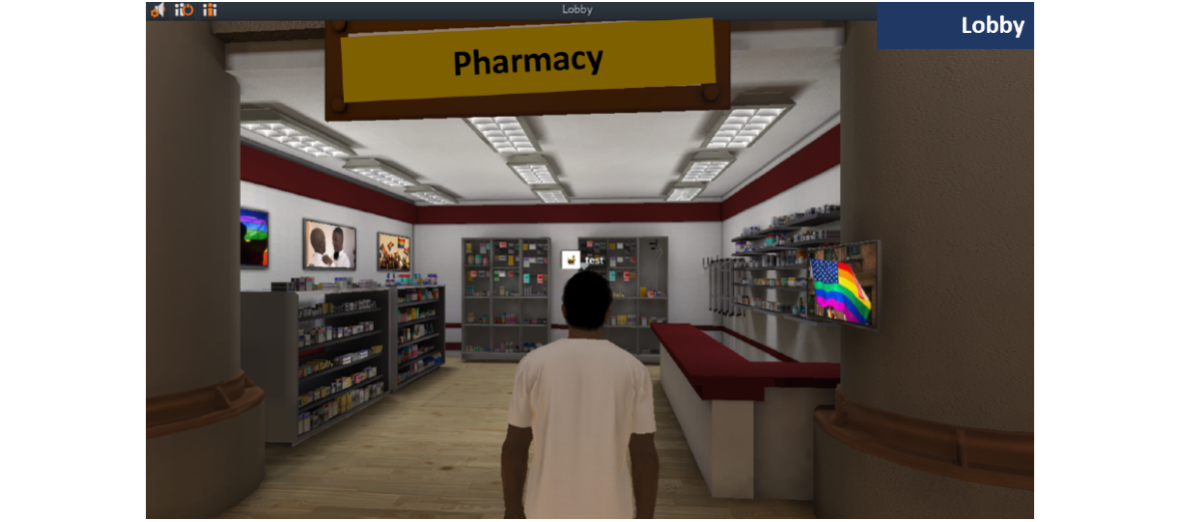
Pharmacy in the lobby.

**Figure 2:**
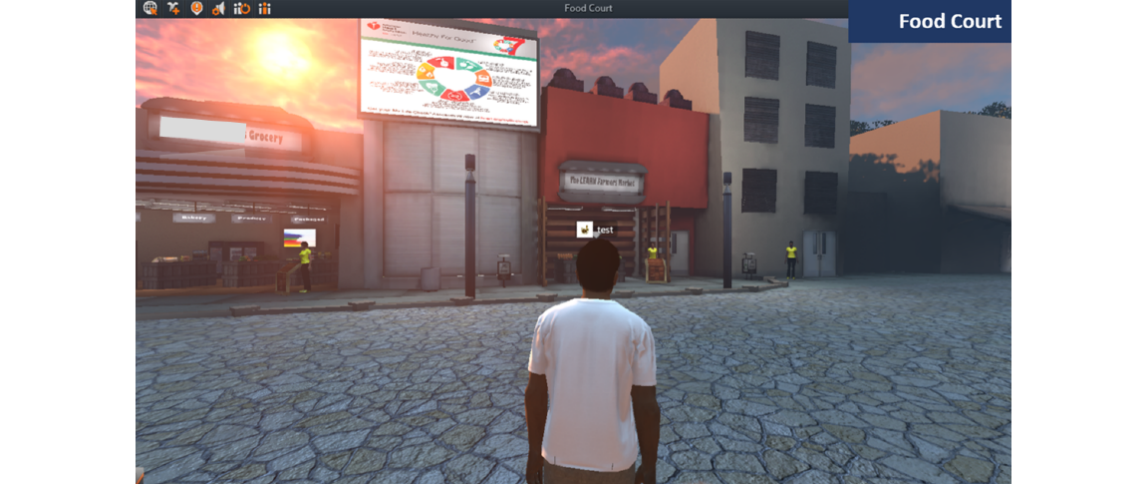
Grocery store in the food court.

The intervention, named Leveraging A Virtual Environment (LEARN) to Enhance Prevention of HIV-related Comorbidities in At-risk Minority Men Who Have Sex With Men, is adapted from a multisite, randomized controlled trial, Diabetes Learning in a Virtual Environment-LIVE [32,66], which was developed and is owned by authors CJ and AV. The LIVE study was a theoretically grounded platform created to support diabetes self-management and facilitate social support using a disease-agnostic environment [38]. In the current proposal, we focus on using a virtual environment for CVD prevention education in persons with HIV. The virtual environment infrastructure is composed of districts where participants can engage with content, such as a pharmacy (Figure 1) or grocery store (Figure 2). Gamification will be used to engage participants and provide motivation to utilize all embedded resources. Participants will interact with other players, complete quests to test their cardiovascular health knowledge, and obtain education on cardiovascular and metabolic comorbidity prevention. Additionally, participants will interact using synchronous voice and text. It is recommended that participants use the virtual environment at least 3 to 4 times a week. We anticipate this will facilitate the establishment of social support in a safe and anonymous online environment [39]. A health educator, trained in the study’s procedures, will facilitate a 1-hour class that will provide cardiovascular health skill-building resources to participants. This will occur monthly over the course of data collection.

### Aim 1

In aim 1, we will qualitatively explore concerns, perceptions about risks, and management and prioritization of HIV-related comorbidities in Zoom meetings with a sample of 15 sexual-minority men of color with HIV. The qualitative interviews are expected to contribute fundamental knowledge to the adaptation of the virtual environment. A qualitative interview guide with 5 open-ended questions will be used. The data obtained will be applied to the resources and to modification of the virtual environment. For example, if hypertension is a prominent concern, we will embed resources focused on reducing high blood pressure. We will also include these and other topics in a curriculum of presentations that the health educator will administer. After completion of aim 1, we anticipate that salient illnesses will be identified and subsequently embedded in the virtual environment. However, because of the diversity of participant ages, we expect to obtain heterogeneous descriptions of comorbidities that are of concern. In the unlikely event that a diverse list of concerns is not obtained, we will embed additional chronic conditions with the highest documented incidence and prevalence in persons of color into the virtual environment. This will help to address any under-identified chronic conditions that persons with HIV may be at risk for.

### Aim 2

In aim 2**,** we will test the feasibility, acceptability, and preliminary effects of the virtual environment to address prevention of CVD comorbidities based on behavioral and psychosocial outcomes in 80 sexual-minority men of color. In order to test this, a waitlist controlled clinical feasibility trial will be conducted. The waitlist design is advantageous for three reasons: (1) recruitment, engagement, and retention will be enhanced by the ability to offer the intervention to all participants; (2) the waitlist control group can receive a version of the intervention improved by addressing any feasibility issues identified in the initial experimental group (ie, the design permits a second iteration of feasibility testing); and (3) the 2-group randomized design yields robust data for estimating group differences in change over time for study outcomes, [67] which will inform a subsequent large-scale grant. Permuted blocks randomization with varying block sizes will be used to ensure allocation concealment and balanced allocation to the intervention and control groups over the study period [68]. Based on augmented reality research by Bailenson and colleagues [69], we anticipate that participants will be more prepared and equipped to apply what they have learned in the virtual environment to everyday life. Although using the virtual environment may seem intuitive, there is a remote possibility that participants may experience technological barriers. As part of the study, we will include a frequently asked questions brochure and contact numbers for technical support.

### Subaim 2a

The purpose of subaim 2a is to characterize the social network structure and behaviors using process data obtained in the virtual environment. We will collect continuous process data from recording and tracking of all interactions within the virtual environment. We expect that the process data will reveal disarticulated patterns, behaviors, and utilization of networks, and show the structure of these networks as they are used by participants in obtaining information [50]. This is important, as the data will be used to identify how social processes affect behavior change [70] and leverage these networks to influence new social norms and behaviors [70]. In the unlikely event that there are technical issues during participant usage, information technology professionals who have designed and worked on LIVE for many years will be available to assist the study team.

### Sample Size Calculation

The purpose of this study is to test the feasibility and acceptability of a virtual environment and obtain preliminary estimates of potential effects on behavioral and psychosocial outcomes in the prevention of cardiovascular comorbidities in persons with HIV. This is not a definitive test of intervention efficacy. Previous research has shown that feasibility studies with a sample size of at least 10 are sufficient to address our aims [30,71]. We believe our sample size of 80 participants (40 intervention subjects and 40 waitlist control subjects) will yield interval estimates of potential intervention efficacy that will be useful for making decisions about next steps (ie, whether to perform more intervention development or launch a larger trial). Power calculations show the planned sample size will produce a 95% CI for mean differences with an expected half-width of 0.45 SD, conveying the precision of preliminary estimates of efficacy.

### Data Analysis

#### Aim 1

Directed content analysis will be used to analyze qualitative data. Directed content analysis is appropriate, as it utilizes a theoretical framework to extend knowledge of data content through a systematic process of coding and identification of categories, themes, and patterns from the interview data [72,73].

#### Measures of Methodological Rigor

Reliability will be demonstrated by providing a detailed description of the study’s purpose, participant sampling, and rationale, as well as the role of the researchers and study team involved [74] and our consistent approach (indicating dependability) to qualitative data collection in aim 1 [75]. Validity will be ensured through member checking [74] of the accuracy and credibility of our findings from the aim 1 interviews. Debriefings will be held after the interviews conclude to determine the validity of our interpretation [74].

#### Aims 2 and 2a

Data will be analyzed using Statistical Package for Social Sciences (SPSS, IBM Corp). Descriptive statistics will describe participant demographics. To determine whether the intervention is acceptable and feasible, the standard will be an acceptability rating of “good” or “very good,” good engagement, and timely completion of study activities in at least 80% of participants. If these standards are met or exceeded, we can conclude that acceptability and feasibility are sufficient for a larger trial. If they are not met, additional formative work on the intervention will be undertaken. CIs for effect sizes will indicate the potential clinical and public health significance and substantiate the need for a larger, more robust clinical trial.

#### Incomplete or Missing Data

To address the potential for incomplete or missing data while maximizing statistical power and reducing the likelihood of bias, several procedures based on differing assumptions about the random nature of the missing data will be implemented [76]. Missing values may be handled by the full information maximum likelihood method, [77] which works by finding model parameters that maximize the likelihood of each case’s observed data [78]. Inverse probability weighting [79,80] and multiple imputation [81-83] will be considered as well. These approaches to handling missing data assume that data are missing at random; that is, missing at random conditionals on values of observed variables. In addition, methods for nonignorable missing data (ie, missing not at random), such as Heckman’s selection model [77,84] and pattern-mixture models [81,85,86], will be used. Sensitivity analyses will be used to determine the degree to which results depend on untestable assumptions.

## Results

The study is part of a 5-year mentored research development award that received funding from the National Heart, Lung, and Blood Institute on September 1, 2019. The first 2 years of the award were developmental. The remaining 3 years will focus on data collection, analysis, dissemination, and an application for a large-scale follow-up grant. We have published 2 manuscripts formative to this research. The first outlined a framework for using eHealth interventions for chronic illness prevention in sexual-minority men of color [39]. The second manuscript was a scoping review assessing nonpharmacologic behavioral and lifestyle interventions to prevent CVD in persons with HIV [87]. Moreover, aim 1 data collection is 87% complete. We expect to complete all data collection for aim 1 on or before April 30, 2022. Recruitment for aim 2 will begin mid-May 2022.

## Discussion

### Principal Aims

We will conduct a waitlist randomized controlled feasibility trial of a virtual environment to provide education on CVD prevention to sexual-minority men of color with HIV who are 30 to 65 years old, which to our knowledge has not been done before. We anticipate that using an anonymous virtual environment for prevention education will have great potential to facilitate health-promoting behaviors over the course of the study and thereafter. Strategies for mitigating challenges were created and will be implemented for all aims, as described above. This study has the potential to inform strategies for chronic illness prevention using a virtual environment in underserved and stigmatized communities, which is advantageous, as technology usage as a means of information seeking and education is standard. In a 2011 survey, 83% of adults with a chronic condition reported using the internet to search for health information [88]. According to a Pew Research poll, 98% of adults 30 to 49 years old and 96% of adults 50 to 64 years old are internet users [89]. Moreover, the proportion of adults who have smartphones (and live in households with incomes less than 30 thousand dollars per year) has increased by more than half [90]. This illustrates the changing demographics and reach of technology use in older age groups and in lower socioeconomic status groups. With the evolving advances in eHealth strategies and uptake in use, virtual environments can become a leading tool for chronic illness risk reduction, as 43% of adults 50 years or older are using technology for health and 38% are using it for skill building [91].

### Limitations

This study is not without limitations. First, the study is not sufficiently powered to detect intervention efficacy. We are testing the feasibility and acceptability of using a virtual environment. Because of this, generalizability is limited. However, our findings will inform any changes or refinements needed for a larger trial. Second, the use of a virtual environment for CVD prevention education may be daunting for persons who are not avid users of technology and may create a barrier to recruitment. We have tried to mitigate this by creating a frequently asked questions brochure. We will also offer orientation prior to the intervention and technical support as needed. Additionally, prior research on older adults suggests that a large proportion of middle-aged to older adults are now playing online games for entertainment and mental sharpness [92]. Third, recruitment may be slow, due to concerns about privacy and confidentiality in a study focused on CVD prevention in persons with HIV. We will reinforce that the virtual environment is an anonymous online space and that all interactions will be via a user-created avatar who uses a pseudonym.

### Conclusion

This study will be the first online virtual environment intervention to address CVD prevention in sexual-minority men of color with HIV. We anticipate that the intervention will be beneficial for CVD prevention education and building peer social supports and will result in changes or modifications in risk behaviors for CVD over time. The ways in which the virtual environment is leveraged can potentially inform strategies on implementation of technology-based behavioral interventions in under-sampled and minoritized communities who are at risk of preventable chronic conditions.

## Appendix

Multimedia Appendix 1 Proposal peer-reviewed summary statement from the National Heart, Lung, and Blood Institute Special Emphasis Panel - K01 Career Development Programs to Promote Diversity in Health Research.

## Abbreviations

CVD: cardiovascular disease
LIVE: Learning in a Virtual Environment
