# Supplementary material for: Cardiovascular Disease Prevention Education Using a Virtual Environment in Sexual-Minority Men of Color With HIV: Protocol for a Sequential, Mixed Method, Waitlist Randomized Controlled Trial
Source: JMIR Res Protoc. 2022 May 17;11(5):e38348. doi: 10.2196/38348 (PMC9157319; doi:10.2196/38348)
Supplement: Multimedia Appendix 1 [file resprot_v11i5e38348_app1.pdf]

SUMMARY STATEMENT
(Privileged Communication)

PROGRAM CONTACT:
Ellen Werner
(301) 435-0077
wernere@nhlbi.nih.gov

Release Date: **04/11/2019**
Revised Date:

Application Number: **1 K01 HL145580-01A1**

Principal Investigator

RAMOS, SILVIA RAQUEL

Applicant Organization: NEW YORK UNIVERSITY

Review Group: **ZHL1 CSR-M (M1)**
National Heart, Lung, and Blood Institute Special Emphasis Panel
K01 Career Development Programs to Promote Diversity in Health Research
AIDS - EXP. REV.

Meeting Date: **03/15/2019**
Council: **MAY 2019**
Requested Start: **07/01/2019**
RFA/PA: **HL19-025**
PCC: **BBCR A**

Project Title: **LEveraging A viRtual eNvironment (LEARN) to Enhance Prevention of HIV-related Comorbidities in at-risk Minority MSM**
SRG Action: **Impact Score:25**
Next Steps: **Visit https://grants.nih.gov/grants/next_steps.htm**
Human Subjects: 30-Human subjects involved - Certified, no SRG concerns
Animal Subjects: 10-No live vertebrate animals involved for competing appl.
Gender: 3A-Only men, scientifically acceptable
Minority: 2A-Only minorities, scientifically acceptable
Age: 3A-No children included, scientifically acceptable

Project Year	Direct Costs Requested	Estimated Total Cost
1	149,904	161,896
2	149,994	161,993
3	149,987	161,986
4	149,813	161,798
5	149,830	161,816
TOTAL	749,528	809,490

ADMINISTRATIVE BUDGET NOTE: The budget shown is the requested budget and has not been adjusted to reflect any recommendations made by reviewers. If an award is planned, the costs will be calculated by Institute grants management staff based on the recommendations outlined below in the COMMITTEE BUDGET RECOMMENDATIONS section.

1K01HL145580-01A1 Ramos, Silvia Raquel

RESUME AND SUMMARY OF DISCUSSION: This is a resubmission K01 Diversity application from Dr. Silvia Raquel Ramos at New York University in the range of excellent to outstanding. This application, discussed with enthusiasm, proposes to use health technology interventions to increase health promotion skills to aid in the prevention of HIV-related cardiovascular and metabolic co-morbidities. The strengths discussed included the thoughtful revisions in response to previous critiques, clear rationale for the research plan, strong candidate, thoughtful career development plan, excellent mentors, and appropriate environment. Concerns were raised about the comparison of different ethnic and racial populations in the research plan as the cardiovascular risk factors of each may be different causing the results to be unclear if the analysis is combined. Further, while the career development plan was considered a strength of the application, some of the panel suggested additional training in clinical trials could have further strengthened the application. Overall, the candidate was considered likely to be competitive for an independent investigator-initiated award upon completion of the proposed studies and the application was rated in the high impact range.

DESCRIPTION (provided by applicant):

LEveraging A viRtual eNvironment (LEARN) to Enhance Prevention of HIV-related Comorbidities in at-risk Minority MSM Project Summary The candidate is an Assistant Professor with expertise in chronic illness and informatics whose overarching career goal is to use health technology interventions to contribute to the achievement of health equity through behavior change and comorbidity prevention in diverse and underserved populations living with Human Immunodeficiency Virus. In order to support this goal and leverage their experiences to date, the Mentored Career Development Award training will focus on three areas: 1) Cardiovascular disease prevention in ethnic/racial MSM, 2) Virtual environment (VE) theory and design, 3) advanced research design methods and innovative analytic approaches. Training will consist of didactic and experiential training, workshops intensives, and attendance at national scientific meetings. The candidate is supported by a distinguished team of mentors who are leaders in their respective fields with successful NIH-funded programs of research in cardiovascular disease and chronic illness prevention, technology-based interventions; and advanced methodological design. The long-term goal of this research is to utilize a VE as a means to increase access to online social supports and increase health promotion skills that can be transferred to the real world and result in the prevention of HIV-related cardiovascular and metabolic conditions. The overall objective of this application is twofold: 1) to receive the training and research experience necessary to become an independent researcher who uses health technology interventions for HIV-related chronic illness prevention, and 2) to address the NIH/NHLBI's call for a diverse early-career scientific workforce (e.g. RFA-HL-19-025). As part of the mentored research, an exploratory sequential wait-list control feasibility clinical trial will be conducted. Aim 1: Explore perceptions about HIV-related comorbidities (i.e., importance, concern) among minority MSM living with HIV. Aim 2: Test the feasibility, acceptability and preliminary effects of a VE to address prevention of HIV- related CVD comorbidities through behavioral and psychosocial outcomes. Aim 2a: Characterize the social network structure and behaviors using process data collected from the VE-based intervention. In Aim 1, 15 qualitative interviews will explore perceptions of HIV-related comorbidity concerns. Aim 2 will be addressed in an iterative multiphase approach. In phase one I will beta test the VE prior to deployment in the proposed clinical feasibility trial. In phase two, I will use the Learning in Virtual Environments (LIVE©) platform to conduct a wait-list control feasibility clinical trial with 80 adult, minority, MSM living with HIV and at-risk for HIV-related comorbidities. While this type of technology has been applied effectively in diabetes and other chronic disease, the ways in which VEs can be used to facilitate knowledge and health promoting behaviors for prevention of HIV-related comorbidities in MSM has not been studied, making this proposal significant and innovative. It is expected that the Mentored Development Award study will represent an early step in the field for prevention of HIV-related comorbidities in ethnic minority MSM and provide the foundation for a R01-funded clinical trial.

CRITIQUE 1

Candidate:	2
Career Development Plan/Career Goals /Plan to Provide Mentoring:	3
Research Plan:	4
Mentor(s), Co-Mentor(s), Consultant(s), Collaborator(s):	3
Environment Commitment to the Candidate:	2

OVERALL IMPACT:
This is a resubmission application from Dr. Ramos who is well qualified for this K award based on her prior research, publication and grant funding history. Her career development goals and training plan appear appropriate. Her research goal of utilizing health technology interventions as a means to contribute to achieving health equity for underserved populations living with HIV and at risk for cardiometabolic disease is in line with NHLBI's goals. The proposed research is innovative in its proposal to use a virtual environment as a means to promote health promoting behaviors and psychosocial outcomes. The environment is excellent and there is evidence of institutional commitment. Enthusiasm is dampened by lack of HIV-specific mentor or consultant and by several concerns regarding details in the research plan.

1. Candidate:
Strengths

- Dr. Ramos is a tenure-track Assistant Professor in the Rory Meyers College of Nursing at New York University. She is well qualified for this K award. Her doctorate was completed at Columbia University and her postdoctoral fellowship at Yale.
- She has experience both in cardiovascular diseases and in HIV as a cardiovascular registered nurse in the cardiac intermediate care and intensive care units and in the cardiac catheterization lab and as a research nurse for the Howard Brown Health Center MACS as well as the Midwest AIDS Training and Education Center's (MATEC) Minority AIDS Initiative.
- She has been successful in obtaining a couple of small grants: An administrative supplement to a parent R01, and pilot funding through the Center for Interdisciplinary Research on AIDS at Yale University.
- She has a good publication record that includes 5 peer-reviewed articles, 4 of them as first author. She has also presented 14 abstracts and has served as an ad-hoc for 3 peer-reviewed journals.

Weaknesses

- None noted.

2. Career Development Plan/Career Goals & Objectives:
Strengths

- The candidate wishes to use health technology interventions as a means to contribute to the achievement of health equity through behavior change and chronic illness prevention in diverse and underserved populations living with HIV and at risk of HIV-related comorbidities. With this goal in mind, the training she seeks within this K award seems appropriate, namely to seek knowledge regarding cardiovascular disease prevention in ethnic/racial MSM; gain experience with Virtual environment (VE) theory and design; and to develop skills in advanced research design, methods and innovative analytic approaches (including randomized clinical trials, social network analysis and data visualization).
- In response to the previous review, the requested duration of K funding has been increased to 5 years.
- It is appropriate that the candidate plans to develop a NIH R01 proposal at the end of the 5 years of K funding.

- A detailed career training table is presented.
- Meeting and evaluation schedule are appropriate.

Weaknesses

- The breakdown of career training details into two tables is somewhat confusing.

3. Research Plan:

Strengths

- The proposed research aligns with the goals and compelling questions of NHLBI – chronic disease prevention and treatment more specifically in HIV and the potential use of virtual learning technology to this field.
- The proposed research is innovative in its use of the virtual environment to provide easy access to health information.
- The research proposes to prevent HIV-related CVD comorbidities in African American and Latino MSM by using gamification of self-management or health behaviors in the VE. Gamification may provide benefit in self-management by allowing anonymity and providing a safe zone; facilitate social networking with other participants and with health educators.
- In response to the previous grant review, a wait-list control design has been incorporated.
- Also, in response to the previous grant review, a section on managing missing data and ensuring credibility of qualitative data has been added to the Data Analysis Section.
- In aim 1, Dr. Ramos will utilize qualitative interviews to explore perceptions of HIV related comorbidity concerns; then in aim 2 phase one, she will beta test the VE prior to deployment in the proposed clinical feasibility trial and in phase 2 utilize the Learning in Virtual Environments (LIVE) platform to conduct a wait-list control feasibility clinical trial with 80 adult, minority MSM living with HIV and at-risk for HIV related comorbidities.

Weaknesses

- The population to be studied are restricted to MSM and excludes women. While MSM are at high risk for HIV, the restriction to this specific group could be argued more effectively (such as potential differences in their perspective on CVD risk compared women).
- Different ethnic groups are likely to have different concerns regarding CVD risk. The reason for lumping blacks and Latinos, particularly without regard to the breakdown between these two ethnic groups in the 15 individuals to be recruited in aim 1 may be a not be ideal.
- For specific aim 1, while it would be valuable to assess the participants views on what is important, it is not clear to this reviewer why the ‘most salient illnesses’ to be included in the VE intervention should be based on participants’ perception of what is important rather than documented research of what intervention is likely to be most beneficial in reducing the risk of CVD in this population.
- Figure 1 seems to be missing.
- For aim 2, critical information is missing as to how often and for how long the participants will be expected to log-on; how often will a health care provider hold group sessions?
- Self-report survey measures are planned at baseline, 3 months and 6 months. Particularly for assessment of knowledge base regarding CVD risk, it is questioned whether frequent administration particularly in a control group will lead to improvement due to a practice effect.

4. Mentor(s), Co-Mentor(s), Consultant(s), Collaborator(s):

Strengths

- Two primary mentors are listed. Dr. Vorderstrasse is Associate Professor and Director of the Florence S. Downs PhD Program in Nursing Research & Theory. Importantly she is an MPI of a NHLBI R01 assessing the utility of virtual environments in diabetes self-management and support. She is an excellent choice for a primary mentor.
- The co-mentor Dr. Kershaw is Professor and Chair of Social and Behavioral Sciences at Yale University School of Public Health, and Director of the Interdisciplinary Methods Core at the Center for Interdisciplinary Research on AIDS at Yale University. Dr. Kershaw is an excellent

choice of a mentor in various implementation science technology, complex designs and advanced analytic techniques.

- The collaborators and consultants are each likely to bring resources to enhance Dr. Ramos' K proposal – Dr. Gwadz, Professor and Associate Dean for Research in NY University for expertise on recruitment of people living with HIV and respondent driven sampling; Dr. Cleland as a collaborator in the planning and analysis of data and Dr. Johnson as a health informatician with interdisciplinary training in health informatics, application of the VE for self-management of chronic illness and data visualization.
- Dr. Ramos has submitted a manuscript with her mentors Drs. Vorderstrasse, Melkus and Kershaw demonstrating a track record of working together.

Weaknesses

- The co-primary mentor Dr. Harmony Reynolds is Associate Professor of Medicine at NYU School of Medicine and Associate Director of the Cardiovascular Clinical Research Center at Langone Health. While likely to provide excellent mentoring in the general area of cardiovascular disease, concern is raised that based on Dr. Reynold's letter of support and the biosketch, Dr. Reynold's expertise does not seem to involve a focus on cardiovascular disease prevention, the focus of Dr. Ramos' K proposal.
- In addition, as this proposal seeks to utilize behavioral intervention specifically in HIV-infected individuals, it is likely that HIV-related behaviors impacting CVD risk (need for full virologic suppression, role of protease inhibitors in dyslipidemia; more recently the potential role of integrase inhibitors in heavy weight gain and depression) may come up as topics of concern. While understanding that Dr. Ramos has previous experience in HIV, it may be a weakness that no mentor who would be able to advise on these aspects are listed.

5. Environment and Institutional Commitment to the Candidate:

Strengths

- The environment is excellent.
- There is evidence of institutional commitment to the candidate in the form of 3 year departmental start-up package valued at over six figures and a commitment by the college of 75% protected time.

Weaknesses

- None noted.

Study Timeline:

Strengths

- Reasonable timeline

Weaknesses

- None noted.

Protections for Human Subjects:

Acceptable Risks and Adequate Protections

Data and Safety Monitoring Plan (Applicable for Clinical Trials Only):

Acceptable

Inclusion of Women, Minorities and Children:

- Sex/Gender: Distribution justified scientifically
- Race/Ethnicity: Distribution justified scientifically
- For NIH-Defined Phase III trials, Plans for valid design and analysis:
- Inclusion/Exclusion of Children under 18: Excluding ages <18; justified scientifically

Training in the Responsible Conduct of Research:

Acceptable

Authentication of Key Biological and/or Chemical Resources:
Acceptable

Budget and Period of Support:
Recommend as Requested
Recommended budget modifications or possible overlap identified:

CRITIQUE 2

Candidate:	1
Career Development Plan/Career Goals /Plan to Provide Mentoring:	1
Research Plan:	1
Mentor(s), Co-Mentor(s), Consultant(s), Collaborator(s):	2
Environment Commitment to the Candidate:	1

OVERALL IMPACT:
This resubmitted K01 Award to Promote Faculty Diversity in Biomedical Research is from Dr. Silvia Raquel Ramos, a nurse scientist and family nurse practitioner, who is currently in her second year as an Assistant Professor at NYU's Rory Meyers College of Nursing. This resubmission application is strong; Dr. Ramos attended to all previous concerns. She expanded her mentoring team to include Dr. Harmony Reynolds, a cardiologist; strengthened her research design for aim #2; added another refereed publication to her total (two more manuscripts have been submitted and are under review); and received funding from an R25 grant. Dr. Ramos's research program focuses on using health technology interventions to contribute to the achievement of health equity through behavior change and comorbidity prevention in diverse and underserved populations living with HIV. Dr. Ramos proposes three aims: 1) To explore perceptions about HIV-related comorbidities (i.e., importance, concern) among minority MSM living with HIV; 2) To test the feasibility, acceptability and preliminary effects of a virtual environment (VE) to address prevention of HIV-related CVD comorbidities through behavioral and psychosocial changes; and 2a) To characterize the social network structure and behaviors of VE participants using process data collected from the VE-based intervention. Dr. Ramos will focus on strengthening her knowledge re: 1) CVD prevention among racial/ethnic diverse MSM living with HIV; 2) virtual environment theory and design; and 3) advanced research methods and analytic approaches. Strengths of the application include applicant dedicated to pursuing an independent research career; strong mentoring team; use of a VE based preventive intervention provided in an anonymous manner to overcome barriers of racism, sexual orientation discrimination and HCP discrimination; strong scientific premise; research strategies to ensure scientific rigor; a well-structured plan for training and strong institutional support. There are few weaknesses in this application, thus based on overall scientific and technical merit, the impact is strong.

1. Candidate:
Strengths
- Dr. Ramos is dedicated to research to improve health equity through behavior change and comorbidity prevention in diverse and underserved populations living with HIV.
- Prior research and experiences provide an excellent foundation for the proposed work.
- 5 refereed publications (plus two others submitted and under review).
- Several presentations at national level professional meetings.
- Recipient of funding from an R25 grant.
- Editorial Board member for Association of Nurses in AIDS care journal

Weaknesses
- None noted.

2. Career Development Plan/Career Goals & Objectives:
Strengths
- Dr. Ramos's proposed training plan will very likely substantially contribute to her development as a scientist and lead to scientific independence.
- Very appropriate targeted skill sets for career development—expertise in CVD prevention in ethnic/racial MSM; virtual environment theory and design; and advanced research methods and analytic approaches (e.g., social network analysis, data visualization).
- Appropriate plans for monitoring and evaluating Dr. Ramos's research and career development progress. Milestones for evaluating progress are described.
- Dr. Ramos will meet biweekly with Dr. Vorderstrasse (Primary mentor) during years 1 and 3 and monthly during years 4 and 5. She will meet with her Co-Primary mentor Dr. Reynolds quarterly and Dr. Kershaw (mentor) monthly in years 1 and 3 and quarterly during years 4 and 5. All mentors and collaborators will meet in person annually.
- Strong letters of recommendation.

Weaknesses
- None noted.

3. Research Plan:
Strengths
- Good scientific premise.
- Dr. Ramos has 3 important aims:
    - To explore perceptions about HIV-related comorbidities (i.e., importance, concern) among minority MSM living with HIV;
    - To test the feasibility, acceptability and preliminary effects of a virtual environment (VE) to address prevention of HIV-related CVD comorbidities through behavioral and psychosocial changes;
    - To characterize the social network structure and behaviors of VE participants using process data collected from the VE-based intervention.
- Dr. Ramos has described strategies to address each aim with a robust and unbiased approach.
- Rationale provided for choice of research designs, selection of samples, recruitment and randomization methods, data entry, handling missing data and analyses.
- Research plans are appropriate for enhancing Dr. Ramos's targeted research skills.
- Sex as a biological variable is addressed.

Weaknesses
- None noted.

4. Mentor(s), Co-Mentor(s), Consultant(s), Collaborator(s):
Strengths
- Dr. Ramos will work with a strong group of mentors and collaborators who have the skills and experiences necessary for her training.
- All mentors are appropriate for the proposed training activities.
- Mentors have strong records of accomplishments.
- There are adequate plans for monitoring and evaluating Dr. Ramos's progress.

Weaknesses
- Unclear how much contact Dr. Ramos will have with Dr. Johnson.

5. Environment and Institutional Commitment to the Candidate:
Strengths
- Strong institutional commitment. Clear commitment that Dr. Ramos will spend 75% of her time on award activities.

- Institutional environment excellent because it has all the resources necessary for Dr. Ramos to carry out her research and career development activities.
- Training support excellent.

Weaknesses
- None noted.

Study Timeline:
Strengths
- Appropriate timeline.

Weaknesses
- Time needed to hire an RA, purchase headsets etc. not accounted for on timeline.

Protections for Human Subjects:
Acceptable Risks and Adequate Protections
- Benefits outweigh any risks. Adequate protections for any potential risk.

Data and Safety Monitoring Plan (Applicable for Clinical Trials Only):
    Acceptable
    - Given small pilot test trial, PI and mentoring team will provide monitoring. Monitoring will occur monthly.

Inclusion of Women, Minorities and Children:
- Sex/Gender: Distribution justified scientifically
- Race/Ethnicity: Distribution justified scientifically
- For NIH-Defined Phase III trials, Plans for valid design and analysis:
- Inclusion/Exclusion of Children under 18: Excluding ages <18; justified scientifically
- Intervention is designed for and only relevant for an adult population.

Resubmission:
- Dr. Ramos addressed all concerns identified in previous review.

Training in the Responsible Conduct of Research:
Acceptable

Authentication of Key Biological and/or Chemical Resources:
Not Applicable (No Relevant Resources)

Budget and Period of Support:
Recommend as Requested
Recommended budget modifications or possible overlap identified:

CRITIQUE 3

Candidate:	1
Career Development Plan/Career Goals /Plan to Provide Mentoring:	1
Research Plan:	3
Mentor(s), Co-Mentor(s), Consultant(s), Collaborator(s):	1
Environment Commitment to the Candidate:	1

OVERALL IMPACT:
In this resubmission application, Dr. Ramos will receive training and research experience necessary to become an independent researcher who uses health technology interventions for HIV-related chronic illness prevention. She proposes three aims but has listed one as aim 2a which are as follows: 1: To

explore perceptions about HIV-related comorbidities (i.e., importance, concern) among minority MSM living with HIV. 2: Using a pilot trial, to test the feasibility, acceptability and preliminary effects of a virtual environment (VE) to address prevention of HIV-related cardiovascular comorbidities through behavioral and psychosocial outcomes, and 2a: To characterize the social network structure and behaviors using process data collected from the VE-based intervention. The candidate is well-trained, and she has assembled an excellent mentoring team. The research plan is good but there are discrepancies in the plan that should be corrected. Overall, this is a strong applicant who proposes an excellent career development plan and a much-needed research topic. She is well positioned to become an independent investigator.

1. Candidate:
Strengths
- Dr. Ramos holds a BSN, MBA, MSN, and a PhD.
- She completed a postdoctoral fellowship.
- She was awarded two grants, (NIH/NICHD R01HD074253 – administrative supplement and NIH/NIHMD R25MH08721 – pilot funding).
- She published her dissertation research in a peer-reviewed journal.

Weaknesses
- She has a limited number of publications.

2. Career Development Plan/Career Goals & Objectives:
Strengths
- The career development plan is excellent with a list of formal coursework in the appropriate areas of cardiovascular disease, VEs, social networks, clinical trials, and grantsmanship.

Weaknesses
- She could benefit from directed readings with respect to clinical trials.

3. Research Plan:
Strengths
- The research plan is innovative and very significant.
- Excellent recruitment plan.

Weaknesses
- In reading the specific aims, there is mention of targeting African American and Latino HIV+ and at-risk MSMs. The research plan, as written, is limited to HIV+ MSM.
- There is some lack of clarity within the approach section such as the description of the trial intervention which is listed prior to the aim which is focused on the intervention.

4. Mentor(s), Co-Mentor(s), Consultant(s), Collaborator(s):
Strengths
- Excellent team with the requisite expertise to support this candidate.

Weaknesses
- None noted.

5. Environment and Institutional Commitment to the Candidate:
Strengths
- Excellent environment.

Weaknesses
- None noted.

Study Timeline:
Strengths
- Very good

Weaknesses
- None noted.

Protections for Human Subjects:
Acceptable Risks and Adequate Protections
Data and Safety Monitoring Plan (Applicable for Clinical Trials Only):
Acceptable

Inclusion of Women, Minorities and Children:
- Sex/Gender: Distribution justified scientifically
- Race/Ethnicity: Distribution justified scientifically
- For NIH-Defined Phase III trials, Plans for valid design and analysis:
- Inclusion/Exclusion of Children under 18: Excluding ages <18; justified scientifically
- Would be helpful to further clarify the justification for exclusion of transgender men.

Resubmission:
- The resubmission is responsive to prior critiques.

Training in the Responsible Conduct of Research:
Acceptable

Authentication of Key Biological and/or Chemical Resources:
Not Applicable (No Relevant Resources)

Budget and Period of Support:
Recommend as Requested
Recommended budget modifications or possible overlap identified:

(End of Reviewers' Comments)

THE FOLLOWING SECTIONS WERE PREPARED BY THE SCIENTIFIC REVIEW OFFICER TO SUMMARIZE THE OUTCOME OF DISCUSSIONS OF THE REVIEW COMMITTEE, OR REVIEWERS' WRITTEN CRITIQUES, ON THE FOLLOWING ISSUES:

PROTECTION OF HUMAN SUBJECTS (Resume): ACCEPTABLE.

INCLUSION OF WOMEN PLAN (Resume): ACCEPTABLE, no women included, scientifically justified.

INCLUSION OF MINORITIES PLAN (Resume): ACCEPTABLE, minorities only, scientifically justified.

INCLUSION OF CHILDREN PLAN (Resume): ACCEPTABLE, no children included, scientifically justified.

VERTEBRATE ANIMAL (Resume): Not applicable.

RESOURCE SHARING PLANS: Not Applicable.

TRAINING IN THE RESPONSIBLE CONDUCT OF RESEARCH: ACCEPTABLE.
Comments on Format (Required):
- In-person courses, online CITI training.

Comments on Subject Matter (Required):

- Subject matter includes: publication and authorship; research misconduct; conflict of interest; ethical treatment of humans as research subjects; good clinical practice; informed consent deficiencies; investigator responsibilities & study team delegation; safety and adverse effects; essential documents; and reporting IRB interaction.

Comments on Faculty Participation (Required; not applicable for mid- and senior-career awards):

- Faculty will participate through mentor/mentee meetings. Topics to be discussed include: scientific integrity re: authorship and publication; protection of human subjects while developing a clinical research protocol; informed consent; and the acknowledgement and rationale for biological variables in research.

Comments on Duration (Required):

- 14 contact hours during post doc; 8 hours completed as a faculty member; will complete another 6 contact hours during award period.

Comments on Frequency (Required):

- Mentoring ongoing through award period; course completion during year three.

AUTHENTICATION OF KEY BIOLOGICAL AND/OR CHEMICAL RESOURCES: Not Applicable.

COMMITTEE BUDGET RECOMMENDATIONS: The budget was recommended as requested.

Footnotes for 1 K01 HL145580-01A1; PI Name: Ramos, Silvia Raquel

NIH has modified its policy regarding the receipt of resubmissions (amended applications). See Guide Notice NOT-OD-14-074 at http://grants.nih.gov/grants/guide/notice-files/NOT-OD-14-074.html. The impact/priority score is calculated after discussion of an application by averaging the overall scores (1-9) given by all voting reviewers on the committee and multiplying by 10. The criterion scores are submitted prior to the meeting by the individual reviewers assigned to an application, and are not discussed specifically at the review meeting or calculated into the overall impact score. Some applications also receive a percentile ranking. For details on the review process, see http://grants.nih.gov/grants/peer_review_process.htm#scoring.

MEETING ROSTER

National Heart, Lung, and Blood Institute Special Emphasis Panel
NATIONAL HEART, LUNG, AND BLOOD INSTITUTE
K01 Career Development Programs to Promote Diversity in Health Research

ZHL1 CSR-M (M1)
03/15/2019

Notice of NIH Policy to All Applicants: Meeting rosters are provided for information purposes only. Applicant investigators and institutional officials must not communicate directly with study section members about an application before or after the review. Failure to observe this policy will create a serious breach of integrity in the peer review process, and may lead to actions outlined in NOT-OD-14-073 at https://grants.nih.gov/grants/guide/notice-files/NOT-OD-14-073.html and NOT-OD-15-106 at https://grants.nih.gov/grants/guide/notice-files/NOT-OD-15-106.html, including removal of the application from immediate review.

CHAIRPERSON(S)

HARDIN, CHRISTOPHER D, PHD
PROFESSOR AND CHAIR
DEPARTMENT OF NUTRITION AND EXERCISE PHYSIOLOGY
UNIVERSITY OF MISSOURI
COLUMBIA, MO 65211

MEMBERS

ARTINIAN, NANCY TRYGAR, PHD
VISITING PROFESSOR
COLLEGE OF NURSING
MICHIGAN STATE UNIVERSITY
EAST LANSING, MI 48824

COLLINS, TRACIE CHIANTI, MD
CHAIR AND PROFESSOR
DEPARTMENT OF PREVENTIVE MEDICINE AND PUBLIC HEALTH
SCHOOL OF MEDICINE
KANSAS UNIVERSITY
WICHITA, KS 67214

DAVIGLUS, MARTHA L, MD
ASSOCIATE VICE CHANCELLOR FOR RESEARCH
DIRECTOR, INSTITUTE FOR MINORITY HEALTH RESEARCH
EDMUND FOLEY PROFESSOR OF MEDICINE
UNIVERSITY OF CHICAGO
CHICAGO, IL 60612

HUBER, SALLY A, PHD
PROFESSOR
DEPARTMENT OF PATHOLOGY AND LABORATORY MEDICINE
VERMONT CENTER OF IMMUNOBIOLOGY AND INFECTIOUS DISEASE
UNIVERSITY OF VERMONT
COLCHESTER, VT 05446

KAPUKU, GASTON K, MD, PHD
ASSOCIATE PROFESSOR
DEPARTMENT OF POPULATION HEALTH SCIENCES
MEDICAL COLLEGE OF GEORGIA
GEORGIA PREVENTION INSTITUTE
AUGUSTA UNIVERSITY
AUGUSTA, GA 30912

MARX, STEVEN O, MD
PROFESSOR
DEPARTMENT OF MEDICINE
DIRECTOR, CARDIOVASCULAR FELLOWSHIP PROGRAM
COLUMBIA UNIVERSITY
NEW YORK, NY 10032

MAYS, RYAN J., PHD
ASSISTANT PROFESSOR
ACADEMIC HEALTH CENTER
SCHOOL OF NURSING
UNIVERSITY OF MINNESOTA
MINNEAPOLIS, MN 55455

OTTERBEIN, LEO E, PHD
ASSOCIATE PROFESSOR OF SURGERY
DEPARTMENT OF SURGERY/TRANSPLANTATION
CENTER FOR LIFE SCIENCES
BETH ISRAEL DEACONESS MEDICAL CENTER
BOSTON, MA 02215

POLLOCK, DAVID M, PHD
PROFESSOR
DEPARTMENT OF MEDICINE AND NEPHROLOGY
SCHOOL OF MEDICINE
UNIVERSITY OF ALABAMA AT BIRMINGHAM
BIRMINGHAM, AL 35233

SHIKUMA, CECILIA M., MD
DIRECTOR AND PROFESSOR
CENTER FOR NATIVE AND PACIFIC HEALTH DISPARITIES
DEPARTMENT OF MEDICINE
JOHN A BURNS SCHOOL OF MEDICINE
UNIVERSITY OF HAWAII AT MONOA
HONOLULU, HI 96816

MAIL REVIEWER(S)

REGENSTEINER, JUDITH G, PHD
PROFESSOR
DEPARTMENT OF MEDICINE
DIVISION OF GENERAL INTERNAL MEDICINE
UNIVERSITY OF COLORADO HEALTH SCIENCES CENTER
UNIVERSITY OF COLORADO AT DENVER
DENVER, CO 80262

SCIENTIFIC REVIEW OFFICER

GARVIN, LINDSAY M, PHD
SCIENTIFIC REVIEW OFFICER
OFFICE OF SCIENTIFIC REVIEW
NATIONAL HEART, LUNG, AND BLOOD INSTITUTE
BETHESDA, MD 20892

EXTRAMURAL SUPPORT ASSISTANT

BROOKS, CAROL
STAFF ASSISTANT
NATIONAL HEART, LUNG, AND BLOOD INSTITUTE
DIVISION OF EXTRAMURAL RESEARCH ACTIVITIES
OFFICE OF SCIENTIFIC REVIEW
BETHESDA, MD 20892

PROGRAM REPRESENTATIVE

ARTEAGA, SONIA S, PHD
PROGRAM DIRECTOR
DIVISION OF CARDIOVASCULAR SCIENCES
NATIONAL HEART, LUNG AND BLOOD INSTITUTE
BETHESDA, MD 20892

CAMPO, REBECCA A., PHD
PROGRAM DIRECTOR
DIVISION OF CARDIOVASCULAR SCIENCES
NATIONAL HEART, LUNG AND BLOOD INSTITUTE
BETHESDA, MD 20892

COADY, SEAN, MS
STATISTICIAN (HEALTH)
DIVISION OF CARDIOVASULAR SCIENCES
NATIONAL HEART, LUNG, AND BLOOD INSTITUTE
BETHESDA, MD 20892

EINHORN, PAULA T., MD
MEDICAL OFFICE/PROGRAM DIRECTOR
DIVISION OF CARDIOVASCULAR SCIENCES
NATIONAL HEART, LUNG, AND BLOOD INSTITUTE
BETHESDA, MD 20792

HUANG, LI-SHIN, PHD
HEALTH SCIENTIST ADMINISTRATOR
DIVISION OF CARDIOVASCULAR SCIENCES
NATIONAL HEART, LUNG AND BLOOD INSTITUTE
BETHESDA, MD 20837

NICASTRO, HOLLY L, PHD
HEALTH SCIENTIST ADMINISTRATOR
DIVISION OF CARDIOVASCULAR DISEASES
NATIONAL HEART, LUNG AND BLOOD INSTITUTE
BETHESDA, MD 20892

PRATT, CHARLOTTE, PHD
HEALTH SCIENTIST ADMINISTRATOR
PROGRAM DIRECTOR
DIVISION OF EPIDEMIOLOGY & CLINICAL APPLICATIONS
NATIONAL HEART, LUNG AND BLOOD INSTITUTE
BETHESDA, MD 20892

REDMOND, NICOLE MD, MS
MEDICAL OFFICER
CLINICAL APPLICATIONS AND PREVENTION BRANCH
NATIONAL HEART, LUNG AND BLOOD INSTITUTE
BETHESDA, MD 20817

REIS, JARED P, PHD
PROGRAM OFFICER
EPIDEMIOLOGY BRANCH
NATIONAL HEART, LUNG, AND BLOOD INSTITUTE
BETHESDA, MD 20892

WANG, WAYNE C., PHD
PROGRAM OFFICIAL
DIVISION OF CARDIOVASCULAR SCIENCES
NATIONAL HEART, LUNG, AND BLOOD INSTITUTE
BETHESDA, MD 20892

WERNER, ELLEN M, PHD
PROGRAM DIRECTOR
DIVISION OF BLOOD DISEASES AND RESOURCES
NATIONAL HEART, LUNG, AND BLOOD INSTITUTE
BETHESDA, MD 20892

WRIGHT, JACQUELINE, PHD
PROGRAM SPECIALIST
DIVISION OF LUNG DISEASES
NATIONAL HEART, LUNG AND BLOOD INSTITUTE
BETHESDA, MD 20892

GRANTS MANAGEMENT REPRESENTATIVE

SINGLETARY, ANNETTE M, MS
GRANTS MANAGEMENT SPECIALIST
OFFICE OF GRANTS MANAGEMENT
NATIONAL HEART, LUNG AND BLOOD INSTITUTE
BETHESDA, MD 20892

OBSERVER

LIDMAN, KARIN FREDRIKSSON, PHD
PROGRAM OFFICER
OFFICE OF RESEARCH TRAINING AND CAREER DEVELOPMENT
DIVISION OF CARDIOVASCULAR SCIENCES
NATIONAL HEART, LUNG, AND BLOOD INSTITUTE
BETHESDA, MD 20892

SHAN, ZHIHONG, MD
SCIENTIFIC REVIEW STAFF (CONTRACTOR)
SCIENTIFIC REVIEW STAFF (CONTRACTOR)
OFFICE OF SCIENTIFIC REVIEW/DERA
NATIONAL HEART, LUNG AND BLOOD INSTITUTE
BETHESDA, MD 20892

Consultants are required to absent themselves from the room during the review of any application if their presence would constitute or appear to constitute a conflict of interest.
